# Giant Cell Aortitis and Tuberculosis: Coincidence or a Causal Link?

**DOI:** 10.7759/cureus.95972

**Published:** 2025-11-02

**Authors:** Said Taharboucht, Asma Azoug, El Djouher Saidoun, Souad Bellaifa, Nacima Djennane

**Affiliations:** 1 Department of Internal Medicine, EHS Salim Zemirli, University of Health Sciences El Moudjahid Youcef El Khatib, Faculty of Medicine, Algiers 1, Algiers, DZA; 2 Department of Anatomic Pathology, CHU Mohamed Lamine Debaghine, University of Health Sciences El Moudjahid Youcef El Khatib, Faculty of Medicine, Algiers 1, Algiers, DZA

**Keywords:** aortitis, giant cell arteritis, giant cell arteritis – tuberculosis coexistence, immunosupressive drugs, large vessel vasculitis, rare association, systemic inflammation, temporal artery biopsy, tuberculosis, vasculitis

## Abstract

Aortitis is a rare inflammatory condition with diverse etiologies. Tuberculosis, though infrequently involved, may coexist with giant cell arteritis (GCA), sharing similar clinical features that complicate diagnosis. Their association is exceptionally rare, presenting a significant diagnostic challenge for clinicians. A 61-year-old man with no prior medical history presented with systemic and inflammatory symptoms, revealing diffuse aortitis associated with both tuberculosis and GCA, the latter confirmed by temporal artery biopsy.

The rare coexistence of tuberculous aortitis and GCA poses a diagnostic challenge, requiring thorough analysis of clinical, biological, histological, and progressive findings.

## Introduction

Aortitis is an inflammation of the aortic wall that can be of infectious, autoimmune, or idiopathic origin [1]. Infectious forms are dominated by syphilis and tuberculosis, although the latter has become rare in industrialized countries. However, it remains a non-negligible cause in countries with high tuberculosis endemicity [2]. The diagnosis of tuberculous aortitis must be made accurately because management differs from that of more common non-infectious aortitis. However, this requirement is often difficult to meet due to the location of the condition. It is a rare entity observed in 1% of aortitis cases [3]. Furthermore, aortitis in giant cell arteritis (GCA) can occur at the onset of the disease or during its course, with a particular tropism for the ascending thoracic aorta [4]. Furthermore, aortitis in GCA can occur at the onset of the disease or during its course, with a particular tropism for the ascending thoracic aorta [4]. GCA is the most common granulomatous vasculitis in the elderly, with an estimated annual incidence of 15 to 25 cases per 100,000 people over the age of 50 [4]. It typically presents with temporal headaches, jaw claudication, a deterioration of general health, and an elevation of inflammatory markers.

Tuberculous aortitis is often characterized by the general symptoms of tuberculosis (fever, night sweats, asthenia, and weight loss) and specific symptoms related to aortic involvement, such as coughing and chest pain.

The association between GCA and tuberculosis is extremely rare, with only a few cases reported in the literature, suggesting a possible pathophysiological link or a simple coincidence. We report the observation of a patient who presented with aortitis of the thoracoabdominal aorta revealing tuberculosis and GCA.

## Case presentation

A 61-year-old man, with no particular medical history, was referred to our department for investigation of a febrile inflammatory syndrome associated with altered general condition. He reported intense asthenia, anorexia, a weight loss of 7 kg, and profuse night sweats. This picture had been evolving in a febrile context for more than five months without joint pain, headaches, or visual disturbances. No history of pulmonary tuberculosis or notion of contact was reported.

On admission, the clinical examination revealed an altered general condition, stable hemodynamic constants, and bilateral and symmetrical arterial pulses present. Cardiopulmonary auscultation was unremarkable, with no cardiac or vascular murmurs. There were no cutaneous signs of vasculitis. The neurological examination was normal, and the ophthalmological examination with funduscopy showed no abnormalities.

The biological assessment showed a marked inflammatory syndrome: ESR at 100 mm, CRP at 119 mg/L, and ferritinemia at 1100 ng/mL (N: 30-300). On CBC, mild normocytic normochromic anemia (Hb: 9.4 g/dL, MCV: 88 fL, MCHC: 32 g/dL) was noted.

Blood cultures, syphilitic, brucella, HIV, HBV, and HCV serologies were negative. EBV, CMV, VZV, and HSV serologies were consistent with past immunization (IgM negative, IgG positive). Protein electrophoresis showed moderate hypergammaglobulinemia at 19 g/L (N: 7-15) and mild hypoalbuminemia at 31 g/L (N: 33-52). The QuantiFERON-TB® test (by the ELISA technique) was positive. The immunological assessment for antinuclear antibodies (ANA) and anti-neutrophil cytoplasmic antibodies (ANCA) was unremarkable (Table 1).

**Table 1:** Laboratory investigation results * indicates values outside the reference range. ANCA, anti-neutrophil cytoplasmic antibodies; ANA, antinuclear antibodies; anti-HCV, hepatitis C virus antibody; AU, arbitrary units; CMV, cytomegalovirus; CRP, C-reactive protein; EBNA, Epstein-Barr nuclear antigen; EBV, Epstein-Barr virus; ESR, erythrocyte sedimentation rate; F, female; Hb, hemoglobin; HBsAg, hepatitis B surface antigen; HIV, human immunodeficiency virus; HSV, herpes simplex virus; M, male; MCHC, mean corpuscular hemoglobin concentration; MCV, mean corpuscular volume; S/CO, signal-to-cutoff ratio; VCA, viral capsid antigen; VZV, Varicella-Zoster Virus.

Parameter	Before Treatment	After 3 Months	After 6 Months	Normal Range	Units
Hematology and Inflammatory Markers					
ESR	100	44	18	0–20 (m) 0–30 (f)	mm/h
Hb	9.4	12.1	14.0	12–16 (f) 14–18 (m)	g/dL
MCV	88	84	88	80–100	fL
MCHC	32	29	32.1	32–36	g/dL
CRP	119	12	≤ 6	≤ 6	mg/L
Ferritin	1100*	801	—	30–300	ng/mL
Serum Proteins					
Gamma Globulins	19*	—	—	7–15	g/L
Albumin	31*	—	35	33–52	g/L
Infectious Disease Serology					
Blood Cultures	Negative	—	—	—	—
Syphilis Serology	0.06	—	—	—	S/CO
Brucella Serology	Negative	—	—	—	—
HIV Serology	0.01	—	—	0.00–1.00	AU/mL
Hepatitis B (HBV)	0.01	—	—	0.00–1.00	AU/mL
Hepatitis C (HCV)	0.01	—	—	0.00–20.00	AU/Ml
Herpesvirus Serology					
EBV					
- IgM anti-VCA	0.27	—	—	0.8–1.1	UI
- IgG anti-VCA	2.86*	—	—	0.8–1.1	UI
- IgG anti-EBNA-1	4.05*	—	—	0.8–1.1	UI
CMV					
- IgM	0.12 (negative)	—	—	0.85–1.00	S/CO
- IgG	> 250*(positive)	—	—	< 6	AU/mL
VZV	IgM (−), IgG (+)	—	—	—	—
HSV	IgM (−), IgG (+)	—	—	—	—
Tuberculosis Screening					
QuantiFERON®-TB Gold	Positive	—	—	—	—
Auto-immune Markers					
ANA	Negative	—	—	< 1/80	—
ANCA	Negative	—	—	< 1/20	—

The electrocardiogram, chest X-ray, and cardiac ultrasound were unremarkable. Arterial Doppler ultrasound of the supra-aortic trunks (SATs) and temporal artery did not show parietal thickening. Angio-computed tomography (CT) of the SATs showed parietal thickening of the proximal segments of the SAT arteries without detectable late enhancement.

**Figure 1:**
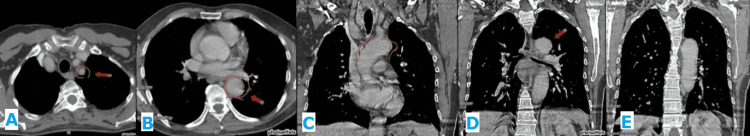
Thoracic computed tomography (CT) scans upon admission (A) Common carotid artery, (B) Ascending aorta, (C) Aortic arch and left subclavian artery, (D) Coronal view of the aortic arch, and (E) Coronal view of the descending aorta

Thoraco-abdomino-pelvic angio-CT described a circumferential and regular hypodense parietal thickening of 3 mm in thickness, weakly enhancing after contrast injection on the large vascular axes: thoracic aorta, carotid arteries, brachiocephalic trunk, subclavian arteries, abdominal aorta, and its iliac branches. These vascular axes were patent and of usual caliber, without detectable stenosis or aneurysm. There were also some small atheroma plaques and a small subcarinal lymphadenopathy of 16×15 mm (Figures 1A-1E).

We were unable to perform a PET-CT scan due to its unavailability in the public healthcare sector and the patient’s financial constraints for accessing it in the private health sector.

**Figure 2:**
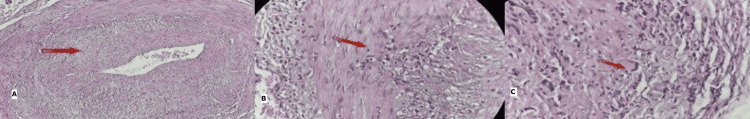
Histology of the temporal artery biopsy The images show signs of granulomatous vasculitis with a thickened, edematous intima (A) surrounded by a medium that's dissociated by a pleomorphic inflammatory infiltrate. This infiltrate is predominantly lympho-histiocytic and is associated with Langhans-type giant cells (C).

The temporal artery biopsy showed granulomatous vasculitis with thickened edematous intima surrounded by a medium dissociated by a polymorphic inflammatory infiltrate predominantly lymphohistiocytic associated with Langhans-type giant cells (Figures 2A-2C).

The diagnosis of tuberculous aortitis associated with GCA was established. We initiated combined antituberculous treatment according to the national protocol: rifampicin (R), isoniazid (H), pyrazinamide (Z), and ethambutol (E) for two months (RHZE), followed by four months of RH, along with weekly methotrexate treatment under close monitoring.

The evolution was favorable with weight recovery, disappearance of general symptoms, and normalization of inflammatory markers.

**Figure 3:**
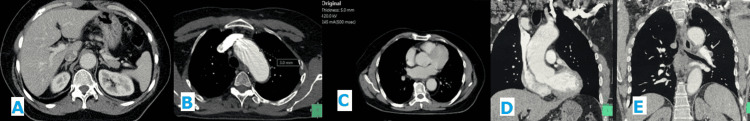
Control thoracic computed tomography (CT) scans A: Axial view/slice of the abdominal aorta showing/objectifying regression of the parietal thickening, measuring 2 mm. B: Axial view/slice of the aortic arch showing/objectifying a non-stenotic circumferential parietal thickening of 3 mm. C: Axial view/slice of the ascending aorta showing/objectifying regression of the parietal thickening, measuring 2 mm. D and E: Coronal image/view of the aortic arch showing regression of the thickening, measuring 2 mm.

Follow-up imaging at six months showed marked regression of the aortic parietal thickening, decreased contrast uptake, and disappearance of the subcarinal lymphadenopathy, confirming regression of vascular inflammation (Figures 3A-3E).

## Discussion

Aortitis is a lesion common to several pathologies, infectious or inflammatory [1]. The association between these two causes is exceptional but not impossible. Its presence poses a real diagnostic challenge due to shared similarities. Our case illustrates the coexistence of GCA, histologically proven, and tuberculosis, suggested by the positive QuantiFERON-TB® test, vascular imaging, and favorable response to antituberculous treatment, despite the inability to obtain any positive culture or specific polymerase chain reaction (PCR) due to the deep location of the aortitis.

Several studies on patients with GCA have shown abnormally low secretion of IFN-gamma, assessed by the QuantiFERON-TB® test [5]. Its positivity in the patient supports the diagnosis of tuberculosis, particularly in the endemic context of our country. It is therefore essential to consider vascular tuberculosis in these patients even in the absence of usual signs of tuberculosis. No link has been demonstrated between tuberculous infection and GCA [6-8], unlike Takayasu's arteritis (TA), where an association has been suggested but remains controversial [9,10].

Existing classification criteria do not allow for the diagnosis of GCA or TA. Early diagnosis of these two vasculitides is complex in clinical practice and can only be established by combining clinical, biological, imaging, and biopsy results, where applicable [11].

The patient presented with GCA without cranial manifestations (absence of headaches, visual disturbances, or jaw claudication) [7] meeting the ACR/EULAR 2022 classification criteria [12]: age over 50 years, biological inflammatory syndrome, aortic involvement on imaging, and giant cells on histology. The diagnosis was therefore established in a context of strongly suspected tuberculosis. TA was ruled out due to the patient's advanced age, absence of arterial stenoses, and histological proof of GCA. The latter mainly affects the aortic arch and thoracic aorta; involvement of the entire aorta is rare [13]. Based on MRI and positron emission tomography (PET) data, more than 50% of patients with GCA have aortic involvement, with a predilection for the thoracic aorta [13,14]. The lesions in our patient are diffuse, involving the ascending thoracic aorta, abdominal aorta, and its iliac branches. This location, the most frequent in vascular tuberculosis [15], suggests a pathological association rather than a simple comorbidity. These two pathologies cause granulomatous vasculitis involving dendritic cells capable of attracting and activating T lymphocytes and macrophages through the production of specific chemokine and cytokine signatures (Th1/Th17 and central role of IFN-γ) [16,17].

In a retrospective Chinese study, among 91 patients with GCA, 20 patients (22.0%) had a history of active tuberculosis and were receiving antituberculous treatment [8].

Two other Mexican patients aged 70 years are described. A man treated for lymph node and hepatic tuberculosis developed GCA one year later. A woman followed for GCA developed systemic tuberculosis three years later [18].

The relationship between infections and GCA has been frequently studied, especially since this condition shows seasonal variation. Patients with GCA experience many more infections, highlighting the potential triggering role of viruses and/or bacteria as triggers [19,20].

In our patient, the final diagnosis was based on the convergence of clinical, radiological, and histological evidence supporting the probable coexistence of tuberculosis aortitis and GCA, suggesting more than a simple coincidence. The combined treatment, antituberculous and methotrexate, allowed for weight recovery, disappearance of general symptoms, and normalization of inflammatory markers.

## Conclusions

This observation illustrates a true diagnostic challenge in internal medicine: the exceptional association between vascular tuberculosis and GCA. In areas where tuberculosis is endemic, this coexistence must be considered, even in the presence of histological evidence (temporal artery biopsy) for GCA. In our case, the diagnosis of tuberculous aortitis was based on clinical data (febrile systemic syndrome), the results of the QuantiFERON-TB® test, imaging showing predominant involvement of the abdominal aorta, and a favorable response to antituberculous treatment. Although the pathophysiological link between these two conditions remains poorly understood, their association necessitates a rigorous diagnostic approach based on a meticulous clinico-radio-biological and histological correlation. A pathophysiological link is likely established between these two conditions, involving common immuno-inflammatory interactions, which suggest more than a simple coincidence. Nevertheless, a rigorous approach guided by clinico-biological and morphological correlation is essential to establish an accurate diagnosis and guide management.

## References

[1] Espitia O, Toquet C, Jamet B, Serfaty JM, Agard C (2024). Aortitis (Article in French). Rev Med Interne.

[2] Revest M, Decaux O, Cazalets C, Verohye JP, Jégo P, Grosbois B (2007). Thoracic infectious aortitis: microbiology, pathophysiology and treatment (Article in French). Rev Med Interne.

[3] Goyal A, Shah I (2017). Aortoarteritis with tuberculosis. J Family Med Prim Care.

[4] Espitia O, Agard C (2013). Aortitis in giant cell arteritis and its complications (Article in French). Rev Med Interne.

[5] Garrot M, Treiner E, Pugnet G (2022). Stimulated interferon-gamma secretion assessed by the Quantiferon® assay: a predictive biomarker of chronicity in giant cell arteritis? (Article in French). Rev Med Interne.

[6] van Timmeren MM, Heeringa P, Kallenberg CG (2014). Infectious triggers for vasculitis. Curr Opin Rheumatol.

[7] Ghesquiere T, Greigert H, Audia S (2019). Study of invariant T lymphocytes during giant cell arteritis (Article in French). Rev Med Interne.

[8] Zhang Y, Wang D, Yin Y, Wang Y, Fan H, Zhang W, Zeng X (2019). Tuberculosis infection in Chinese patients with giant cell arteritis. Sci Rep.

[9] Kumar Chauhan S, Kumar Tripathy N, Sinha N, Singh M, Nityanand S (2004). Cellular and humoral immune responses to mycobacterial heat shock protein-65 and its human homologue in Takayasu's arteritis. Clin Exp Immunol.

[10] Carvalho ES, de Souza AW, Leão SC (2017). Absence of mycobacterial DNA in peripheral blood and artery specimens in patients with Takayasu arteritis. Clin Rheumatol.

[11] Keser G, Aksu K (2019). Diagnosis and differential diagnosis of large-vessel vasculitides. Rheumatol Int.

[12] Ponte C, Grayson PC, Robson JC (2022). 2022 American College of Rheumatology/EULAR Classification criteria for giant cell arteritis. Arthritis Rheumatol.

[13] Finucci Curi P, Sattler ME, Chaves M (2023). Extensive aortic involvement in giant cell arteritis. Reumatol Clin (Engl Ed).

[14] Tyagi S, Safal S, Tyagi D (2019). Aortitis and aortic aneurysm in systemic vasculitis. Indian J Thorac Cardiovasc Surg.

[15] Marcu DT, Adam CA, Mitu F (2023). Cardiovascular involvement in tuberculosis: from pathophysiology to diagnosis and complications-a narrative review. Diagnostics (Basel).

[16] Sneller MC (2002). Granuloma formation, implications for the pathogenesis of vasculitis. Cleve Clin J Med.

[17] Deng J, Younge BR, Olshen RA, Goronzy JJ, Weyand CM (2010). Th17 and Th1 T-cell responses in giant cell arteritis. Circulation.

[18] Alba MA, Mena-Madrazo JA, Flores-Suárez LF (2013). Giant cell arteritis and disseminated tuberculosis: presentation of two cases. Scand J Rheumatol.

[19] Russo MG, Waxman J, Abdoh AA, Serebro LH (1995). Correlation between infection and the onset of the giant cell (temporal) arteritis syndrome. A trigger mechanism?. Arthritis Rheum.

[20] Greigert H, Genet C, Ramon A, Bonnotte B, Samson M (2022). New insights into the pathogenesis of giant cell arteritis: mechanisms involved in maintaining vascular inflammation. J Clin Med.

